# OOCDB: A Comprehensive, Systematic, and Real-time Organs-on-a-chip Database

**DOI:** 10.1016/j.gpb.2023.01.001

**Published:** 2023-01-12

**Authors:** Jian Li, Weicheng Liang, Zaozao Chen, Xingyu Li, Pan Gu, Anna Liu, Pin Chen, Qiwei Li, Xueyin Mei, Jing Yang, Jun Liu, Lincao Jiang, Zhongze Gu

**Affiliations:** 1 School of Life Science and Technology, Southeast University, Nanjing 210096, China; 2 School of Biological Science & Medical Engineering, Southeast University, Nanjing 210096, China; 3 School of Computer, Data & Information Sciences, University of Wisconsin-Madison, Madison, WI 53706, USA

**Keywords:** Database, Organ, Mathematical model, Citation map, Organs-on-a-chip

## Abstract

**Organs-on-a-chip** is a microfluidic microphysiological system that uses microfluidic technology to analyze the structure and function of living human cells at the tissue and **organ** levels *in vitro*. Organs-on-a-chip technology, as opposed to traditional two-dimensional cell culture and animal models, can more closely simulate pathologic and toxicologic interactions between different organs or tissues and reflect the collaborative response of multiple organs to drugs. Despite the fact that many organs-on-a-chip-related data have been published, none of the current **databases** have all of the following functions: searching, downloading, as well as analyzing data and results from the literature on organs-on-a-chip. Therefore, we created an organs-on-a-chip database (OOCDB) as a platform to integrate information about organs-on-a-chip from various sources, including literature, patents, raw data from microarray and transcriptome sequencing, several open-access datasets of organs-on-a-chip and organoids, and data generated in our laboratory. OOCDB contains dozens of sub-databases and analysis tools, and each sub-database contains various data associated with organs-on-a-chip, with the goal of providing researchers with a comprehensive, systematic, and convenient search engine. Furthermore, it offers a variety of other functions, such as **mathematical modeling**, three-dimensional modeling, and **citation mapping**, to meet the needs of researchers and promote the development of organs-on-a-chip. The OOCDB is available at http://www.organchip.cn.

## Introduction

Two-dimensional cell culture [1], [2] and animal models [3] are commonly used in traditional clinical drug research. There is, however, a structural distinction between monolayer cells *in vitro* and natural tissues. Furthermore, traditional *in vitro* cell culture models cannot fully compare the interactions of various organs and tissues [1], [4], [5]. Therefore, the efficacy of drug screening cannot be tested accurately [6], [7]. Furthermore, due to interspecific differences between humans and animals, several drugs that pass animal model screening may fail in clinical trials due to toxicity to humans or a lack of curative effect [8]. Therefore, there was an urgent need for new technology to complete the *in vitro* construction of pathologic and physiologic models*,* and thus organs-on-a-chip technology was developed. Organs-on-a-chip are *in vitro* microfluidic microphysiological systems that mimic the structural and functional properties of human tissues and organs [4], [9]. Microprocessing technology, cell culture, biomaterials, and other methods are used to create it on a microfluidic chip [10], [11]. It not only performs high-resolution, real-time imaging analysis of the structure and function of living human cells at the tissue and organ levels *in vitro* [11], but it also eliminates the uncertainty caused by complex metabolism in traditional pharmacokinetic studies [2], [12].

Various organs-on-a-chip technologies have been developed in recent years [13]. These organ-on-chips contain various organ-specific cell types [4] that can be used to simulate corresponding organs. Lung chips [14], [15], liver chips [16], cornea chips [10], and heart chips [17] are a few examples. The disease modeling data of various organs have seen explosive growth as a result of the advancement of this technology. Furthermore, the complexity of human organs-on-a-chip structure, measurement connotation, and the rapid development of large amounts of data impedes the use of organs-on-a-chip data.

Among established databases, the University of Pittsburgh's microphysiology systems database (MPS-DB) is the only one dedicated to organs-on-a-chip [18], [19]. It can successfully collect and use data from organs-on-a-chip and makes significant contributions to the development of a related database [20]. This database, however, has some limitations. First, MPS-DB contains a large amount of organ-on-a-chip model data, and the organ-on-a-chip data collection is extensive. However, content for organ-on-a-chip-related data, such as literature, patents, and other publicly available data, is relatively scarce. Therefore, data richness is insufficient. Second, the MPS-DB data cannot be completely downloaded. Only the models' brief data are available for download; however, the pictures and detailed descriptions of the models are not; thus, insufficient data are available. Furthermore, some public databases, such as PubChem [21], NCBI [22], DrugBank [23], ChEMBL [24], and BRD (https://brd.nci.nih.gov/brd/), are available for organs-on-a-chip research. However, due to a lack of relevance and standardized methods and management systems for large multidimensional and heterogeneous organs-on-a-chip data, they are insufficiently useful in organs-on-a-chip research. We created the organs-on-a-chip database (OOCDB) in response to the urgent need to better prepare for future research and provide a comprehensive and searchable database for scholars in relevant fields, we have developed the OOCDB.

The OOCDB is a standardized system that contains a large number of charts, literature, and patent data. Furthermore, the database content is updated on a regular basis. Every month, relevant literature, patents, and other information are gathered to keep researchers up to date on the latest developments and research hotspots. Furthermore, when compared with other databases, OOCDB has the following benefits. (1) This database is divided into sections, which include literature, patents, drugs, poisons, chemicals, and other information, with each section containing a variety of open-access data. The database's content is extensive, and the data are detailed. Furthermore, the OOCDB contains a large amount of lab-generated data, which supplements the public data and forms the data source of the OOCDB. (2) Our database handles data related to organs-on-a-chip, analyzes the author, publishing time, and publishing unit of the data, and provides researchers with an analysis of the most recent research findings. (3) Our database can also offer free online tools for researchers, such as mathematical modeling, by allowing them to upload their own data or data of interest. These data will be analyzed using tissue enrichment analysis to determine the organ of gene enrichment, and they will thus correspond to their own experimental results and serve as a research reference. Therefore, OOCDB can further promote data exchange and sharing of on-chip organs, which is critical for accelerating on-chip organ research and overcoming complex diseases.

## Database content and methods

### Data collection and integration

**Figure 1:**
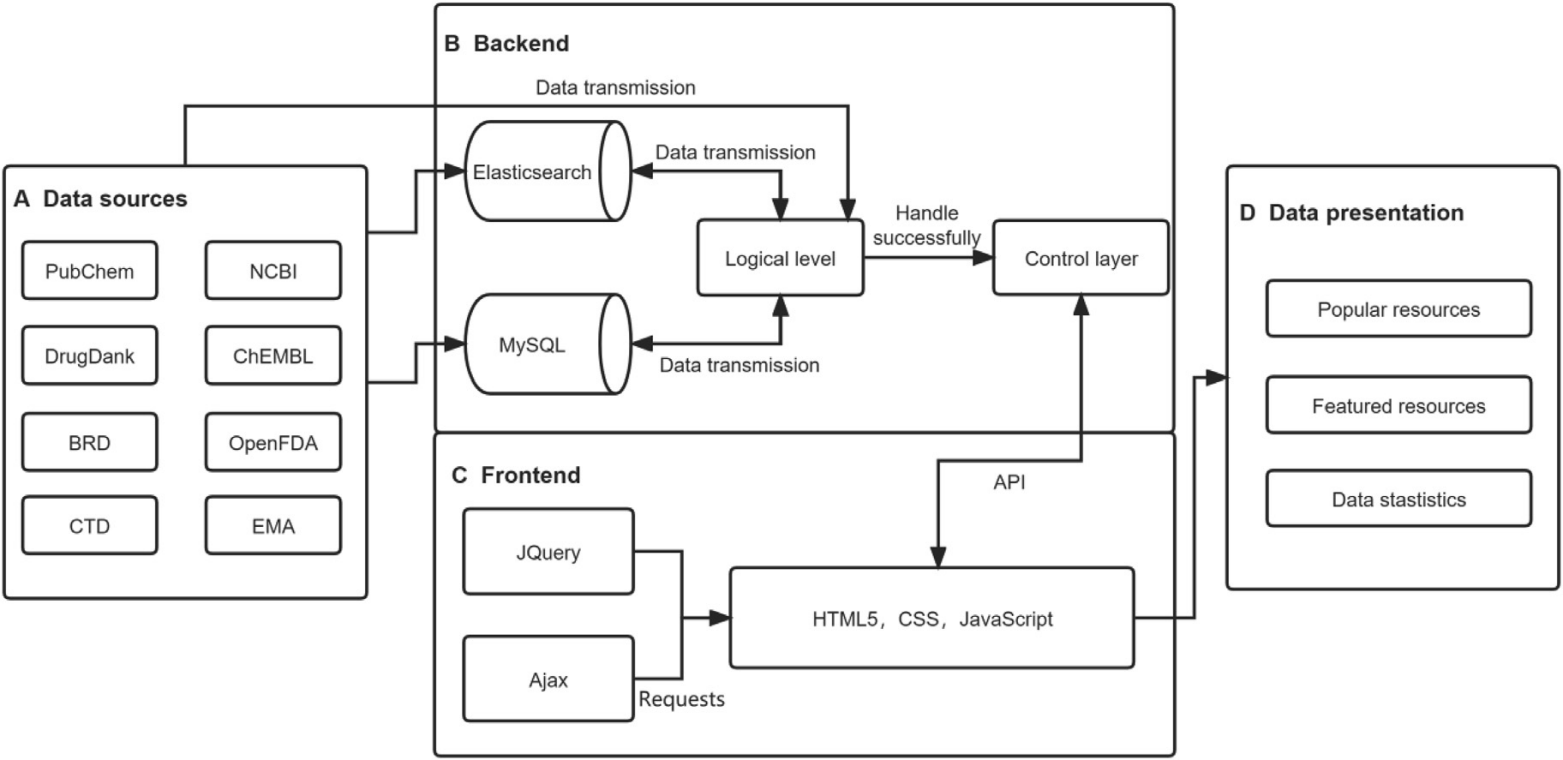
**The main construction principle of OOCDB** **A.** The OOCDB's data sources primarily include PubChem, NCBI, DrugBank, ChEMBL, BRD, OpenFDA,CTD, and EMA. **B.** The OOCDB's backend. Elasticsearch and MySQL process the data based on the logical level, and the results are then transferred to the control layer. **C.** The OOCDB's frontend. To create frontend web pages, backend data are transferred via the API and combined with JQuery and Ajax via HTML5, CSS, and JavaScript. **D.** The OOCDB data presentation. Following frontend processing, the data are primarily classified as popular resources, featured tools, and data statics. OOCDB, organs-on-a-chip database; NCBI, National Center for Biotechnology Information; BRD, Biospecimen Research Database; CTD, Comparative Toxicogenomics Database; EMA, Europan Medicines Agency; API, application program interface.

It is the main framework, interaction, and logic of our OOCDB, as illustrated in Figure 1. To create a high-quality OOCDB, a standardized data collection and organization process is used. We used “organ-on-a-chip”, “organs-on-a-chip”, “organ on chips”, “organ chips”, and “organoid chip”. “Heart chip”, “liver chip”, “vascular chip”, “skin chip”, “lung chip”, “intestinal chip”, “neural chip”, “tumor chip”, and “reproductive system and embryo chip” keywords were retrieved from PubMed (https://www.ncbi.nlm.nih.gov/pubmed/), NCBI [22], BRD, and Google patent-related publications (https://patents.glgoo.top/). The data were filtered manually by reading the abstracts and full text, and the data from publications not related to organ chips were excluded. There were 107,873 relevant literature data, 16,841 histology data, and 11,915 patent data collected in total. Furthermore, we extracted information on drugs, toxins, compounds, and devices from literature, patents, and histology data. We obtained relevant open-access data using keywords from DrugBank, ChEMBL, CTD [25], and EMA (https://www.ema.europa.eu/en), and provided links to the original websites for non-open-access data. A total of 14,020 drug records, 17,329 poison records, 4,986,258 compound records, and 160 device records were collected.

We imported the collected data into the control layer for analysis and integration, as shown in Figure 1B. The literature data were classified and processed according to the different organs and cancers studied and then entered into the organ and cancer research databases, respectively. At the same time, the authors, citations, and cited relationships were processed to create the citation data. We used the citation data to create a citation map, integrating and interconnecting over 100,000 papers. Furthermore, we used the Elasticsearch tool (https://www.elastic.co/cn/elasticsearch/) to connect the literature and drug libraries, filtering out drugs that appear in the literature and linking them to drugs in the drug library, so that drug data can be accessed directly through the literature and literature data can be accessed through the drugs. We categorized patent data by country and checked the patent application and expiry dates, providing the option to filter patents by date. We sorted the statistics for histology data by the organ studied and provided a distribution map of the organs studied. In addition, we processed the histology data and integrated it with the literature data using the Elasticsearch tool. Finally, rather than being separate databases, the databases in OOCDB were interconnected and based on the MySQL database system.

### Database construction

To create the database index, we used the Elasticsearch tool. We set the primary key of the MySQL database as the article title, patent title, drug name, poison name, and other data to avoid duplicate data because the primary key of a database is unique. As shown in Figure 1C, the data processed by the control layer are transferred to the front end via the application programming interface (API). For web page construction, the database employs the jQuery framework, HTML5, CSS, and JavaScript technologies, and requests are handled via Ajax. Finally, as shown in Figure 1D, the OOCDB database was created, which included popular resources, featured tools, and publication statistics.

### Database interface

**Figure 2:**
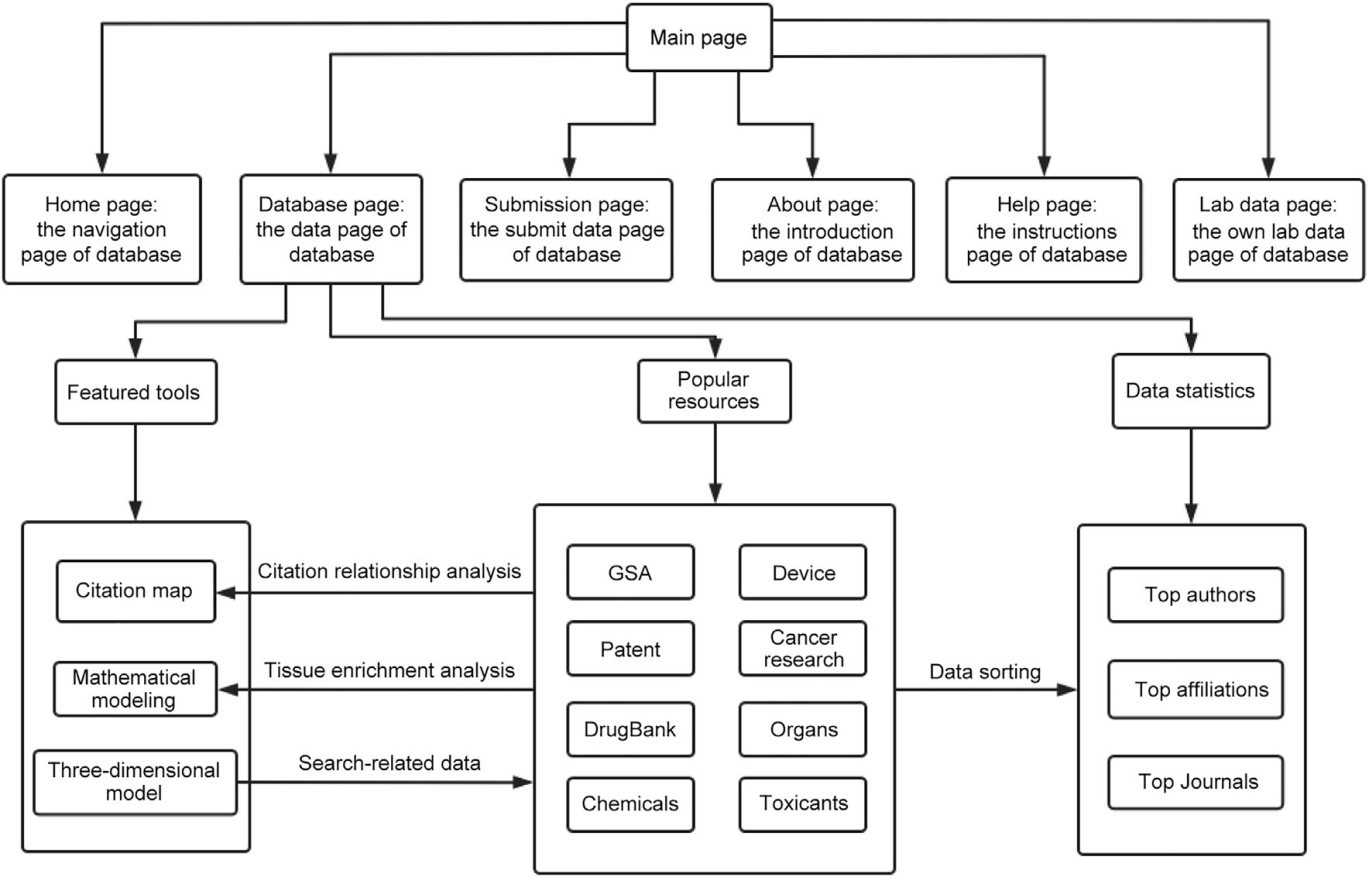
**The main framework of OOCDB** OOCDB's six navigation bars are the home page, the database page, the submission page, the about page, the help page, and the lab data page. The database page is the most important part of OOCDB. The highlighted tools, popular resources, and publication statistics are all linked. Popular resources primarily store basic data, including GSA, DrugBank, devices, organs, patents, cancer research, chemicals, and toxicants. In addition, featured tools and publication statistics rely primarily on popular resources for analysis and processing. GSA, Genome Sequence Archive.

Figure 2 depicts the OOCDB architecture. There are six navigation bars, eight sub-databases, and three featured tools for data analysis processing and related statistical analysis in the OOCDB, which can provide data associated with organs-on-a-chip literature and patents, as well as brief information about drugs, toxins, compounds, and their applications in organs-on-a-chip. The home page, database page, submission page, about page, help page, and lab data page are the six website navigation bar entries, as shown in Figure 2. The database page is the most important part of OOCDB, and it contains popular resources, featured tools, and publication statistics. The most popular resources are the Genome Sequence Archive (GSA) [26], DrugBank, devices, organs, patents, cancer research, chemicals, and toxicants. Each sub-database has its own set of unique data. Three-dimensional (3D) models, mathematical modeling, and citation map are examples of featured tools that have achieved innovative advancement. In the publication statistics, the top authors, top affiliation, and top journals sections reflect trending research topics and the most recent discoveries.

A simple search in OOCDB will yield the following results: (1) relevant data on organs-on-a-chip, such as literature, patents, and histology data; (2) information about drugs, devices, organs, patents, chemicals, toxicants, data from the lab itself, and other organs-on-a-chip-related data; and (3) names of famous experts, institutions, and journals in the field of organs-on-a-chip, which can be used for organs-on-a-chip hotspot analysis. OOCDB provides a variety of online tools in addition to easy access to required information. Users can, for example, use mathematical models for tissue enrichment analysis, choose the database's own standard database or a database provided by the user for comparison, and thus identify the organs enriched by the genes of interest. Furthermore, users can submit their organs-on-a-chip-related research results in our submission interface to enrich the database content in order to collect more organs-on-a-chip-related data.

## Results

**Figure 3:**
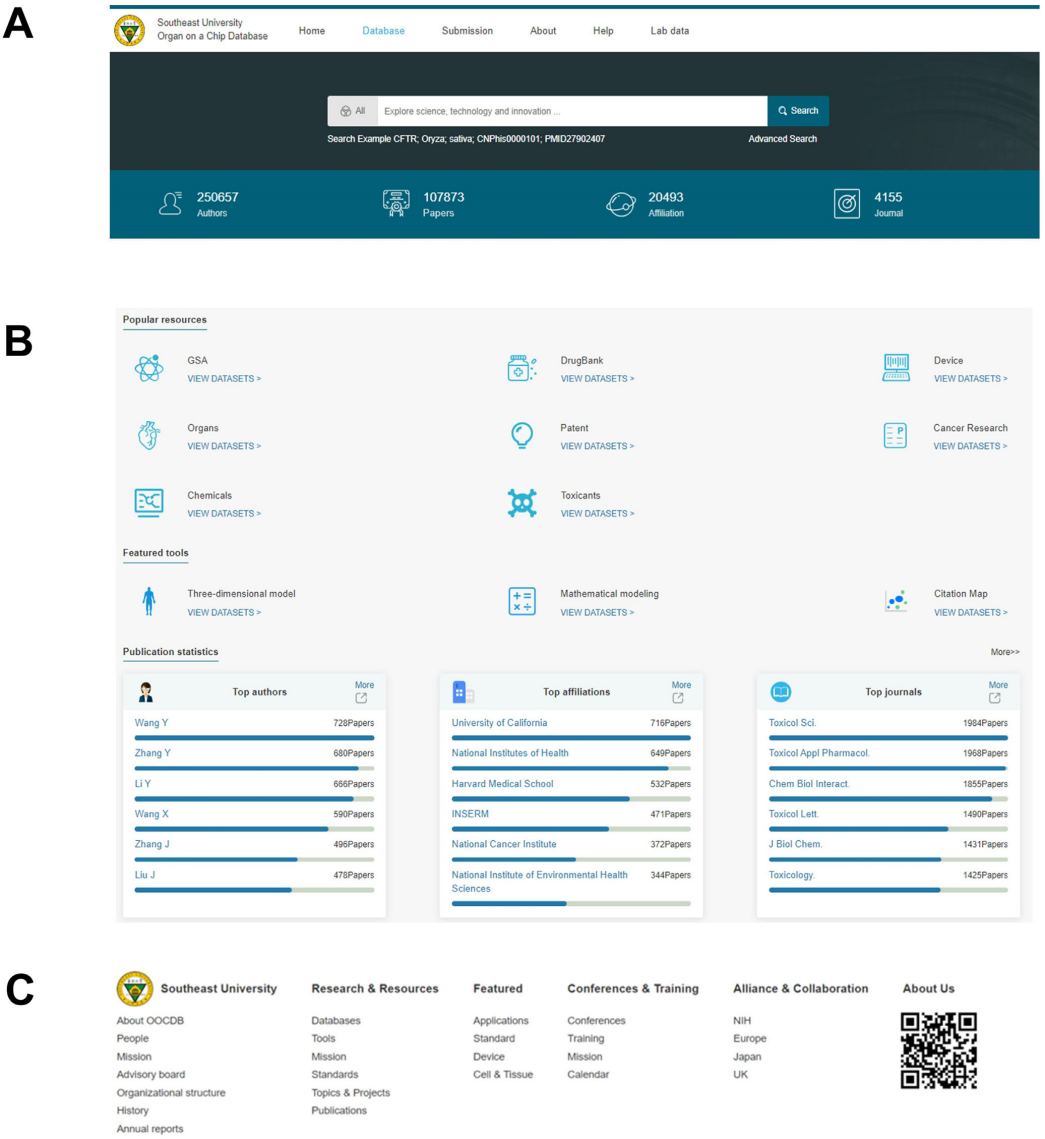
**The screenshot of the main interface of OOCDB showing four parts of the website** **A.** The OOCDB search box and data were introduced. **B.** The database's main body, including featured tools, popular resources, and publication statistics. **C.** The basic information about the OOCDB.

The OOCDB page is divided into three sections. First, there is a search box at the top of the page (Figure 3A). To switch to other proprietary databases, such as literature, drugs, and GSA, click the “All” button. Users enter an advanced search by clicking the advanced search switch located beneath the search bar. A keyword search on literature, drugs, GSA, and patent databases is called an advanced search. The OOCDB statistics are located at the bottom of the search page. OOCDB currently has 122,000 academic papers related to organs-on-a-chip, including over 4000 academic journals and 20,000 affiliations involving 25,000 researchers and experts in the field. Second, our database's core components are popular resources, featured tools, and publication statistics (Figure 3B). GSA, DrugBank, devices, organs, patents, cancer research, chemicals, and toxicants are among the most popular resources. View datasets direct users to the desired sub-database and query its contents. A 3D model, mathematical modeling, and citation map are among the tools available. By entering the corresponding section, the user can process the data. Top authors, top affiliations, and top journals are all included in publication statistics. Users can inquire about their information by clicking more. Finally, the page displays basic information about the database, such as the resource, database features, and developer information (Figure 3C).

### Popular resources

#### GSA database

The GSA database contains a total of 16,841 cases of raw microarray and transcriptome sequencing data. These data are classified according to human organs and tissue types, such as lung, muscle, skeleton, nervous system, genetic system, and soft tissue. The database website contains the exact number of microarray and transcriptomic sequencing. In addition, the years of publication of organs-on-a-chip-related literature are tracked. Furthermore, the statistical results revealed that research data on organs-on-a-chip has been increasing in recent years, and organs-on-a-chip is gradually becoming a trending research area. Data in GSA can be searched in OOCDB using ordinary and advanced search functions. When using the standard search, keywords can be used to retrieve information. Advanced search features include “and”, “or”, and “not relationships”, which can be used to search for multiple keywords that have relationships between them. It also includes access number, title, experience type, organization name, department, and lab, all of which can be searched individually. The search result includes status, title, organization(s), experience type, summary, overall design, contributor(s), platforms, samples, and relations. These files are saved in the OOCDB and can be downloaded in soft, minimal, and text formats.

#### DrugBank database

**Figure 4:**
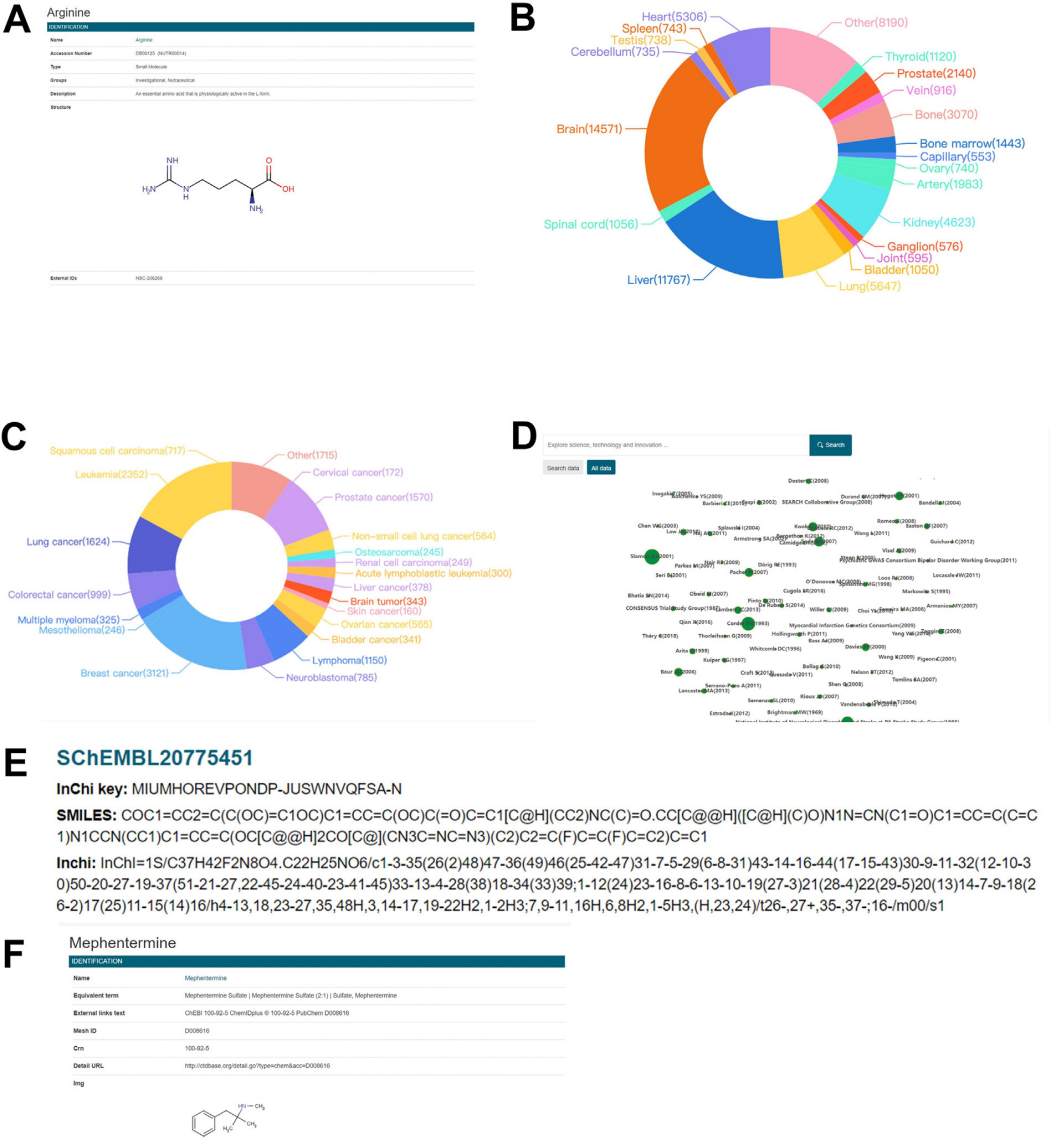
**A brief introduction to the sub-databases** **A.** Drug detail interface in the DrugBank database. **B.** Organ distribution map in the organ database. **C.** Cancer distribution map in the cancer research database. **D.** Citation map of all data. **E.** Chemical detail interface in the chemical database. **F.** Toxicant detail interface in the toxicant database.

The DrugBank database contains information on 14,020 different types of drugs that are linked to organs-on-a-chip. The database, as shown in Figure 4A, provides drug information such as name, accession number, structure, chemical formula, synonyms, unique ingredient identifier (UNII), international chemical identifier (InChi), InChi key, simplified molecular input line entry system (SMILES), weight, drug entry, and other drug information to assist users in locating drug-related information. Furthermore, there are two types of drug searches: basic and advanced. Furthermore, the advanced search features “and”, “or”, and “not relationships”, and information can be found by entering a drug name, type, chemical abstracts service (CAS) number, description, UNII, or groups. Furthermore, if this drug is used in organs-on-a-chip-related experiments, OOCDB provides literature data on its use.

#### Device database

We first presented a distribution map of 160 studies on organs-on-a-chip devices in the device database (Table 1). Users can access the literature database by clicking on the desired organs-on-a-chip devices in the distribution map. The database provides an overview of organs-on-a-chip devices, including fabrication materials, manufacturing technology, fluid drive and control in microfluidic chips, and signal detection.

**Table 1:** **Number of studies for each device in the device database**

**Material/method**	**Number of studies**
TCPS	1
PDMS	52
Hydrogel	22
Membrane	60
Three-dimensional printing	1
Microfabrication	18
Machining	4
Pouring	2

*Note*: TCPS, tissue culture polystyrene; PDMS, polydimethylsiloxane.

#### Organ database

The organ database categorizes studies based on organ names, resulting in 97 different categories, each corresponding to an organ. By comparing these category keywords to the organs-on-a-chip literature that we downloaded, we were able to screen and classify 77,628 relevant documents (Table 2). After that, the top 20 organs with the highest frequency were chosen for a graphic display. As shown in Figure 4B, most studies on organ microarray in the organ database were about the brain (15,974), liver (11,844), and lung (5758), which is also the research hotspot of organ microarray. OOCDB offers full-text downloads of these related studies, as well as reference and citation maps. A citation map is created with the reference and citation, and the size of the circle indicates the author's influence in this field. The larger the circle, the greater the influence of the authors, making it easier for researchers to find experts in the field.

**Table 2:** **Number of studies for each organ in the organ database**

**Organ**	**Number of studies**
Brain	15,974
Liver	11,844
Lung	5758
Heart	5445
Kidney	4659
Bone	3111
Prostate	2159
Artery	2074
Bone marrow	1455
Other	25,149

#### Patent database

The patent database was created by screening out 54 keywords related to organs-on-a-chip, searching for patents on Google, and retrieving a total of 11,915 patents. These patents are downloaded into our database and classified based on country. As shown in Table 3, the United States (4028), China (3301), and Japan (1444) hold a large number of patents related to organs-on-a-chip and thus have a significant advantage in the field. Furthermore, OOCDB offers full-text retrieval and the ability to download relevant patents. Users can narrow their search scope in the OOCDB by date and country, and they can also retrieve patents in the advanced search by title, author, abstracts, description, patent number, and country expiry date. Therefore, users can easily download the patents they require.

**Table 3:** **Number of patents for each country in the patent database**

**Country**	**Number of patents**
United States	4028
China	3301
Japan	1444
European Patent Office	669
France	352
Russia	348
South Korea	295
Germany	281
Australia	256
Other	941

#### Cancer research database

The cancer research database is similar to the organ database. Using 87 cancer keywords, 30,158 articles were found, and a chart displaying the top 20 cancers frequently mentioned in papers was created (Figure 4C). Breast cancer (3189), leukemia (2386), and lung cancer (1701) are frequently mentioned in the cancer database, and organs-on-a-chip are widely used in these cancers (Table 4). This database, like the organ database, includes a citation map to help you find the most authoritative scholars in the fields of organs-on-a-chip and cancer (Figure 4D).

**Table 4:** **Number of studies for each cancer in the cancer research database**

**Cancer**	**Number of studies**
Breast cancer	3189
Leukemia	2386
Lung cancer	1701
Prostate cancer	1593
Lymphoma	1168
Colorectal cancer	1004
Neuroblastoma	793
Squamous cell carcinoma	719
Ovarian cancer	571
Non-small cell lung cancer	622
Other	16,412

#### Chemical database

The chemical database contains data on 4,986,258 compounds related to organ microarrays. The compounds are listed alphabetically*.* The compound database provided data such as InChi key, SMILES, and InChi (Figure 4E). Users can easily find the structures and properties of related compounds.

#### Toxicant database

The toxicant database contains data on 17,329 different types of toxicants. They are alphabetically arranged and can be retrieved in the order of the alphabet. Furthermore, users can enter the name of the toxicant in the search box and be taken directly to the toxicants interface. Information on various toxicants, including name, equivalent terms, other database numbers, CAS registry number, structure image, and external links, is provided in the toxicant detailed interface (Figure 4F)*.* If some toxicant properties are not found in OOCDB, users can use external links to enter the special toxicant database for a query. At the same time, we combined the toxicant database with the literature database on organs-on-a-chip. If the toxicant is used in organ-on-a-chip research, the relevant literature is listed below.

### Featured tools

The featured tools in OOCDB present the most important characteristics of organs-on-a-chip, and the user can obtain these data. A 3D model, mathematical modeling, and citation map are all part of the feature resource.

#### 3D model

**Figure 5:**
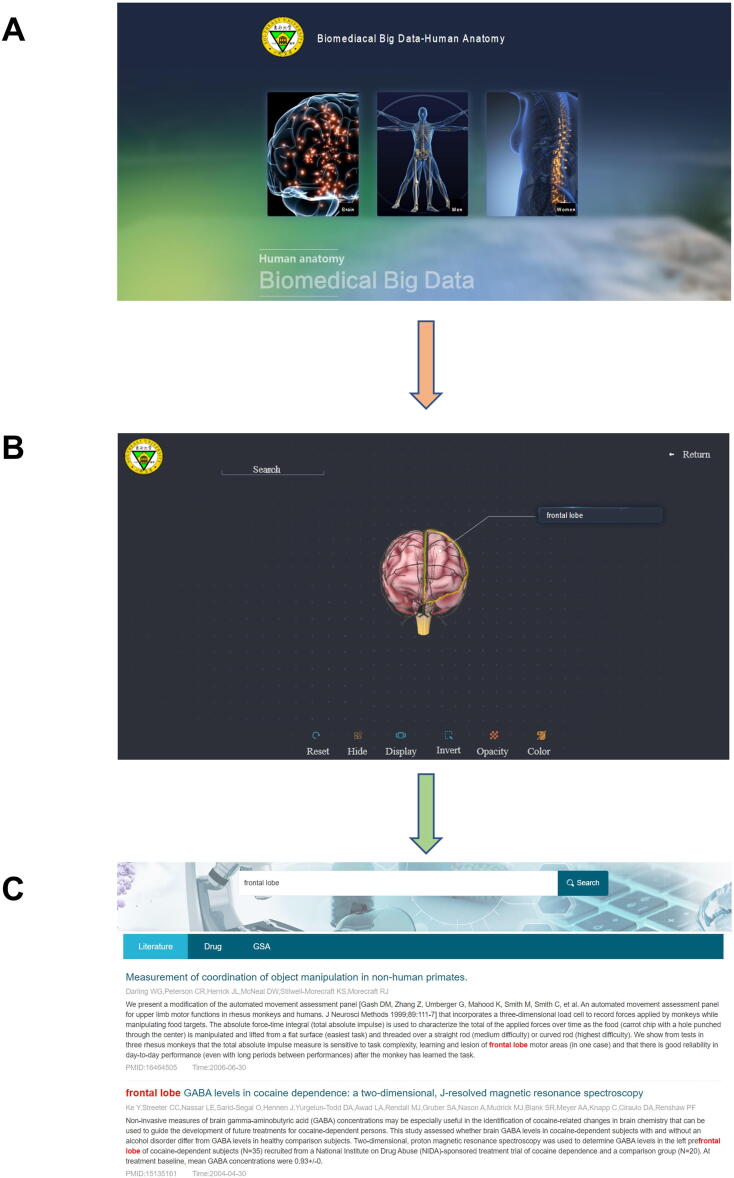
**Steps of searching for related literature by the organization on a chip study** **A.** Enter the chip study sub-database organization. **B.** Enter the 3D model you are looking for. **C.** To access the relevant literature, click on the organ's name.

A 3D model is a tool for understanding the combination of organs-on-a-chip data in OOCDB and human organs at the anatomical level. Users can navigate and find the target organ by navigating in the 3D anatomical atlas for the human brain male and female bodies. A visual window displays actual human anatomical structures (Figure 5). There are three parts to this: a human brain model, a male anatomical model, and a female anatomical model. Figure 5 shows that the database contains high-quality human brain models, male anatomical models, female anatomical models, as well as internal structures viewed from a surgical perspective. Users can enter the corresponding model by clicking the image, and then clicking it again to enter the corresponding organs. Users can navigate several anatomical layers in the human body or brain in each model. They can enter any organ with the help of an anatomical map. There are 111, 2984, and 3000 different types of brain organs, male organs, and female organs, in each model, respectively. Users can get the corresponding organ name by clicking the corresponding position of the organ in the model, and then by clicking the name of the organ, users can view omics data, literature data, and drug data of the organ in OOCDB to find detailed information on the organs-on-a-chip and the current research status. It helps researchers find relevant information and advances the development of organs-on-a-chip technology.

#### Mathematical modeling

**Figure 6:**
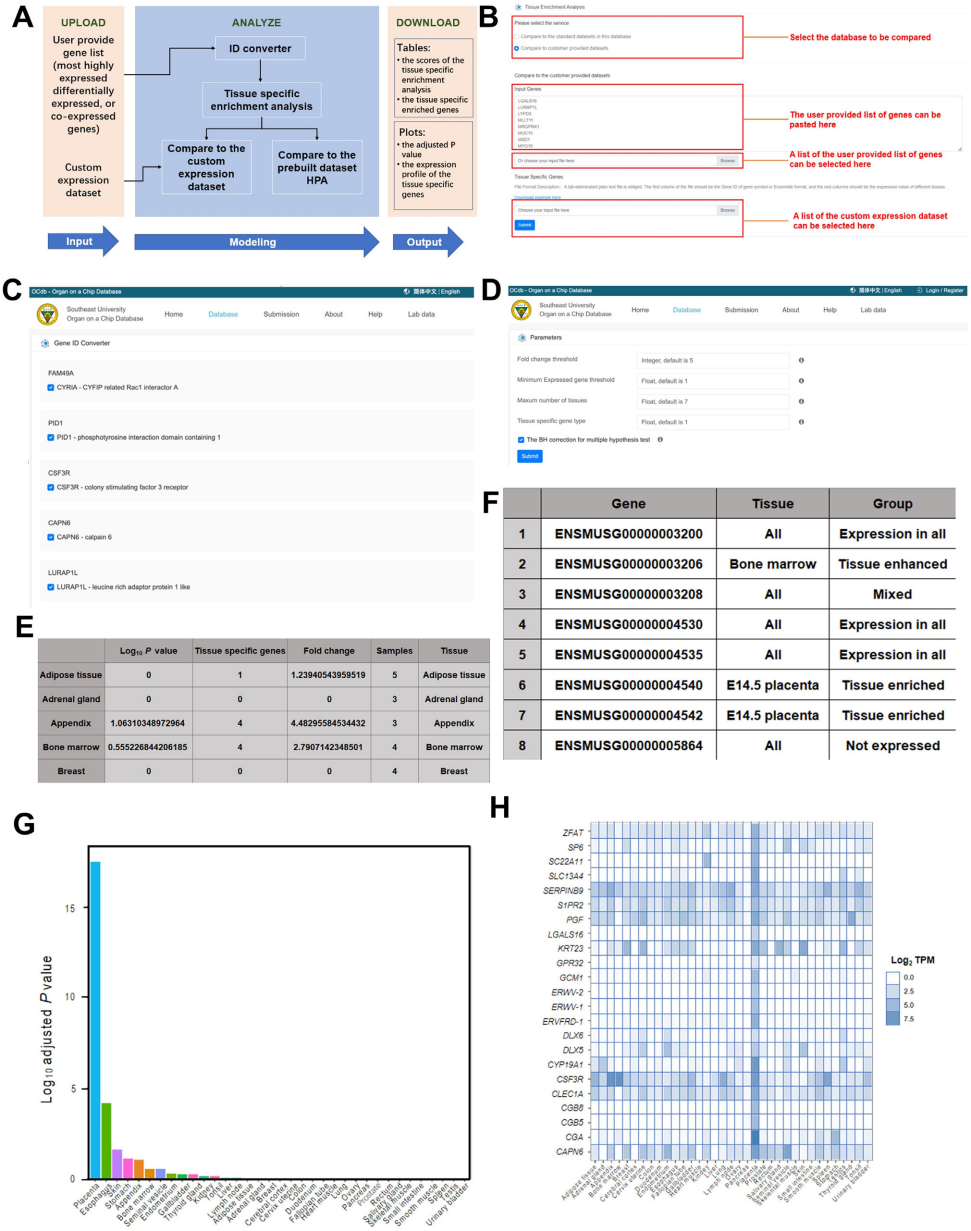
**Tissue enrichment analysis performed in the mathematical model** **A.** The workflow of the mathematical model includes input, modeling, and output. **B.** The input of the mathematical model. First, the user selects a dataset. Second, the user provided a gene list. Third, the user selected a file containing a list of genes. Finally, the user chose a file from the custom expression dataset. **C.** Tissue-specific enrichment analysis. Program checked. The program validated the genes uploaded by the user to ensure that they are recognizable for enrichment analysis, and the users can select the genes based on the id conversion results. **D.** Fold change threshold, minimum expressed gene threshold, the maximum number of tissues, and tissue-specific gene type are all available on the parameters page. **E.** Enrichment analysis scores include log_10_*P* value, tissue-specific gene number, fold change, samples, and tissue type. **F.** The genes that are overrepresented. **G.** The plots are the log_10_ bar plots (*P* value). **H.** The heat map of the expression profile of the tissue-specific genes, giving the log_2_ TPM value of each gene. TPM, transcripts per kilobase million.

Most users are concerned about whether their own data derived from an *in vitro* organs-on-a-chip platform are consistent with the organ *in vivo* and how similar they are. Therefore, we included a section on mathematical modeling that gives users a useful tool for estimating consistency between *in vitro* data from their organs-on-a-chip platform and gene expression data from real human organs. This section currently includes TissueEnrich, a tool for calculating the enrichment of tissue-specific genes in a set of input genes using the hypergeometric test [27]. This tool is embedded in a web server and has an easy-to-use graphical user interface. The tissue specificity of cells cultured *in vitro* in an organs-on-a-chip platform can be tested by the user. This tool's workflow consists of three steps: input, modeling, and output (Figure 6A). The user uploads the gene list of the most highly expressed genes, differentially expressed genes, or co-expressed genes to the web server for the input step. The gene list can be typed directly into the tool's web page or saved as a file (Figure 6B). In addition, in the following step, the user can provide an expression dataset of interest for further tissue-specific enrichment analysis (Figure 6C). After clicking the submit button, a program will automatically check the identity document (ID) of the genes uploaded by the user to confirm that the genes are recognizable for the enrichment analysis. Furthermore, users can select genes based on the results of the ID conversion (Figure 6C). The confirmed genes are sent to the enrichment analyzer, which compares the gene list with prebuilt datasets or a user-supplied custom expression dataset. The user must choose whether to use the Benjamini–Hochberg correction for multiple hypothesis testing, which is recommended, when comparing to prebuilt datasets. For defining tissue-specific gene expression, two prebuilt human datasets are provided: human protein atlas (HPA) [28] and genotype-tissue expression (GTEx) [29]. If you use the custom expression dataset, the server will take you to the parameters setting page (Figure 6D). When all of these settings are complete, the server will begin the analysis. Tables and plots will be used to display the results. The tables contain the enrichment analysis scores, such as the log_10_ *P* value, tissue-specific gene number, fold change, samples, and tissue type (Figure 6E), as well as the enriched genes (Figure 6F). The plots are the log_10_ *P* value bar plot (Figure 6G) and the heat map of the tissue-specific gene expression profile (Figure 6H). All tables and plots are available for users to download.

#### Citation map

**Figure 7:**
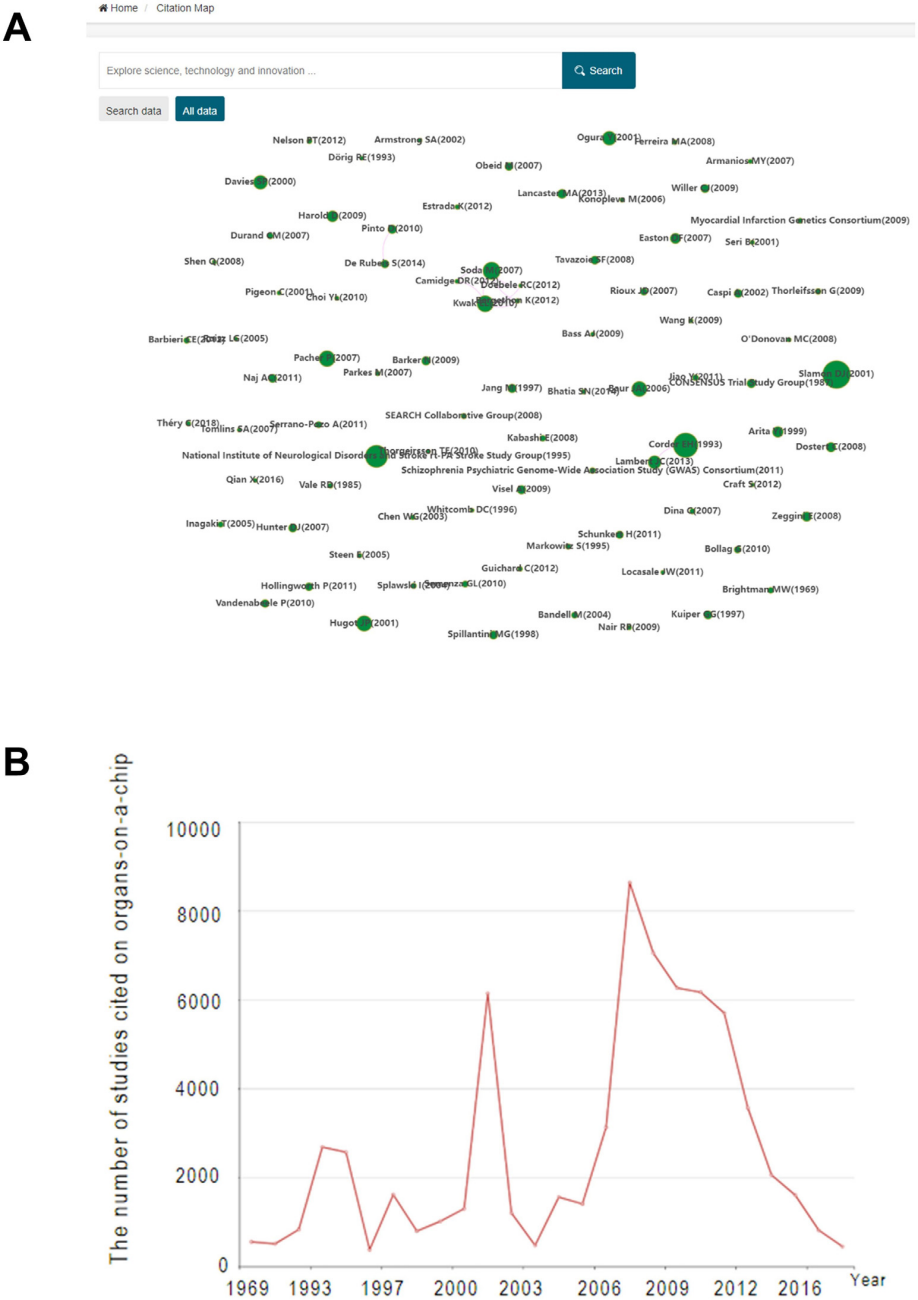
**Introduction of citation map** **A.** A graph of authors who have received a high number of citations in their field. **B.** Change of cited literature in the field over time.

A citation map is an OOCDB tool for analyzing the citation situation in organ chips. Users can use the citation map's search box to look for a field keyword. It will search the organs-on-a-chip literature database for authors based on the keywords entered by users and visually present them. Furthermore, users can visualize the entire dataset by clicking on all data. Figure 7A shows that the size of the circle indicates the number of papers cited by that author in the field, with the larger circle indicating the author's number of cited articles in the field. Furthermore, the citation relationship of each author in the field, if any, is expressed in the citation map by connecting the lines. Figure 7B also includes a trend graph of publications in the field, illustrating the change in the number of studies cited on organs-on-a-chip over time. Therefore, the citation map tool can be used to identify research hotspots in the field of organs-on-a-chip and promote the development of organs-on-a-chip-related fields.

### Lab data

**Figure 8:**
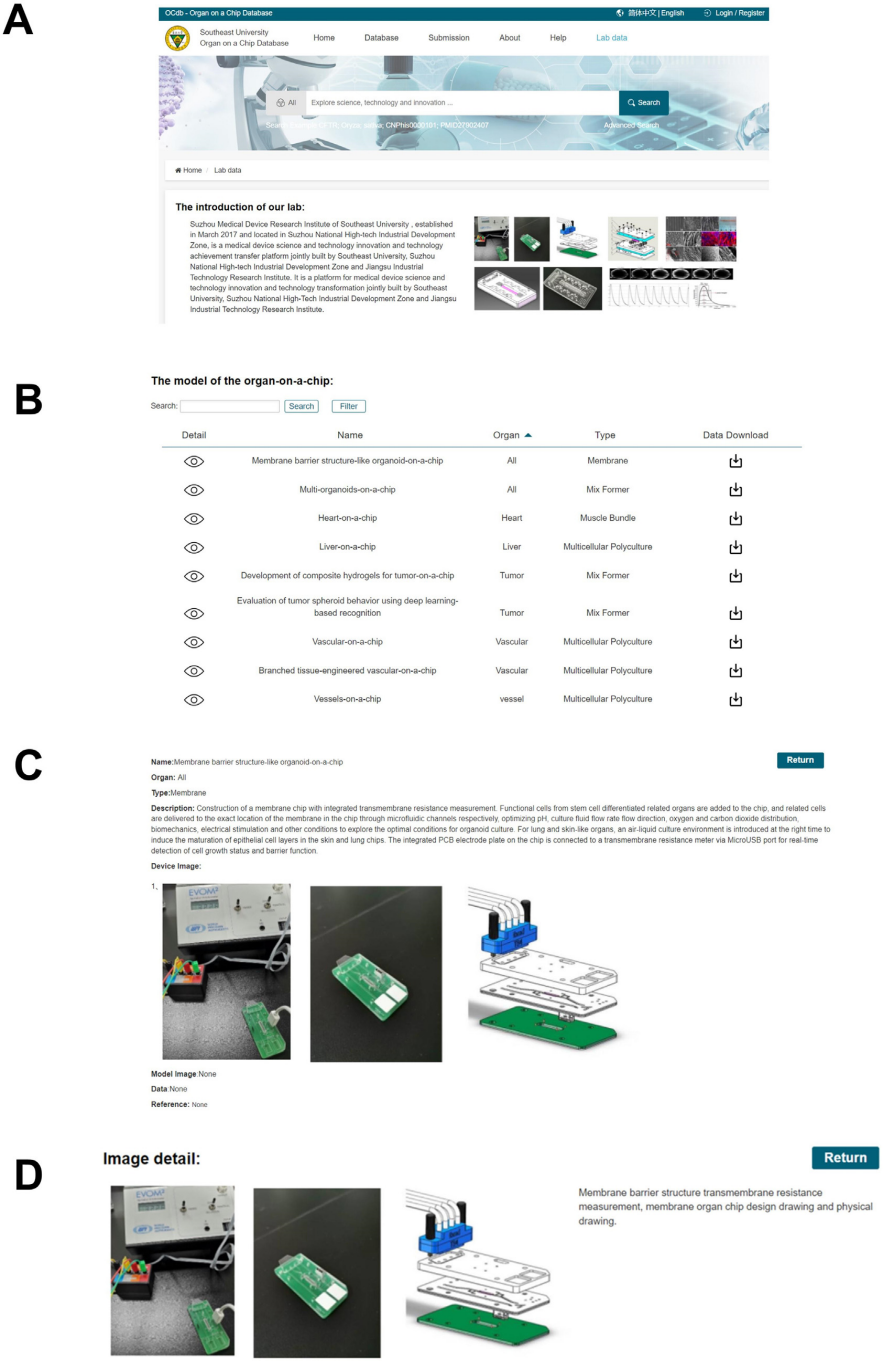
**Introduction of in-house data** **A.** The introduction of organ-on-a-chip data from our lab. **B.** Major organ model data from our lab. **C.** Detailed information on organs-on-a-chip models. **D.** The image details of the organ chip.

Figure 8 depicts the lab data section, which includes data and models of organs-on-a-chip from laboratories. At the moment, it primarily involves our research institute's results as a demonstration, such as blood vessels-on-a-chip, epidermis-on-a-chip, heart-on-a-chip, and tumor-on-a-chip, and it will include data from our chip's end users, collaborators, and other labs (Figure 8A). The organs-on-a-chip model includes search, filter, and download capabilities. Users can search directly for relevant organs-on-a-chip data or filter data by organ and/or simulation method to quickly and easily find the data they need. Meanwhile, we provided a download function that allowed users to directly download the data they needed (Figure 8B). Users can also view detailed organs-on-a-chip data by clicking “detail,” which provides detailed information for each organ-on-a-chip. We have divided our own lab data into six fields: name, organ, type, description, device image, and model image, so that researchers can browse and study the data measured or recorded from each chip in detail (Figure 8C). Furthermore, users can view the details of an image by clicking on a device or model image to learn more about the organ chip (Figure 8D).

### Publication statistics

Furthermore, OOCDB compiles authors, institutions, and journals. Users can view the number of published papers, citation frequency, and publication trend of top authors, top affiliations, and top journals in the current period by selecting different time periods. Users can sort by count or citation and select authors, institutions, and journals to read.

### Case study: using OOCDB to study hepatocellular carcinoma

OOCDB makes it simple and quick to conduct organ-on-a-chip research. In comparison to other open public databases, OOCDB focuses on organ-on-a-chip, allowing researchers to quickly find relevant information. OOCDB can provide more comprehensive data than MPS-DB. Here is an example of what OOCDB can do for hepatocellular carcinoma.

**Figure 9:**
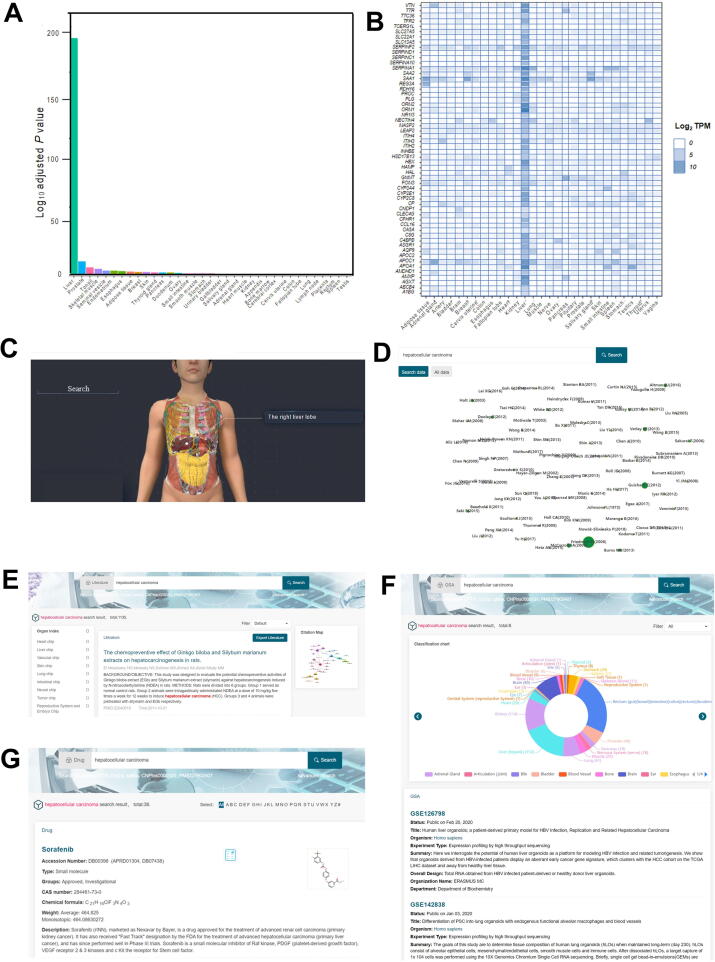
**Application of OOCDB in hepatocellular carcinoma research** **A.** Results of tissue enrichment analysis of 318 hepatocellular carcinoma-associated genes. **B.** Heatmap of 318 hepatocellular carcinoma-associated genes for specificity analysis. **C.** 3D model for the localization of the liver. **D.** Search results for hepatocellular carcinoma in citation map. **E.** Hepatocellular carcinoma search results in the literature database. **F.** GSA database search results for hepatocellular carcinoma. **G.** Search results for hepatocellular carcinoma in the drug database.

Tissue enrichment analysis for genes associated with hepatocellular carcinoma can be performed using a mathematical model and a dataset of tissue-enriched genes from 12 nontumor tissues [30]. We used the method of Li et al. [30] to perform enrichment analysis on these 318 genes. The information was processed by entering the gene symbol and selecting the human protein atlas. These genes were mostly enriched in the liver, which matches the findings of Li et al. [30] (Figure 9A). These findings demonstrated the mathematical model's dependability. The mathematical model identified genes that were highly enriched in the liver, such as *SERPINA1*, *APOA1*, *ORM1*, and others, laying the groundwork for future experiments (Figure 9B). As shown in Figure 9C, we can also use the 3D model to quickly locate the liver and the organs adjacent to it in the body and perform a quick search for these organs. Furthermore, OOCDB has a citation map function, as shown in Figure 9D, which shows the results of a citation map search for hepatocellular carcinoma, which includes information on experts in the field. OOCDB provides a large amount of information on literature, histology, and drugs for hepatocellular carcinoma (Figure 9E−G) so that researchers can easily and quickly retrieve the information they need to conduct their research.

## Conclusion and future plans

Organs-on-a-chip technology has been widely concerned and developed rapidly with the rapid development of modern science and technology. Organs-on-a-chip is a new platform for *in vitro* cell culture that was created by combining microfabrication technology and 3D cell culture. This method has the advantages of portability, high throughput, and *in vivo* microenvironment simulation over traditional modeling methods. Furthermore, it has broad application prospects in disease pathogenesis research and drug screening. Furthermore, it can provide an integrated and systematic solution for life sciences and medical research, as well as play a critical role in a variety of fields, including life science and medicine. Furthermore, because organs-on-a-chip is a new technology, the data generated by it is currently in the “blowout” stage. Several organs-on-a-chip-related studies are constantly published, and organs-on-a-chip research is maturing. Therefore, a database related to organs-on-a-chip must be established.

OOCDB is a unified platform (science as a service) that provides scientific research communities with organs-on-a-chip biological big data sharing and application services. It is built on big data, artificial intelligence, and cloud computing technology, and it offers data services specialized for organs-on-a-chip data archiving, computational analysis, knowledge search, sharing services, and visualization.

We completed the corresponding data archiving, annotation, analysis, and visualization work in OOCDB, resulting in dozens of sub-databases and analysis tools. Furthermore, OOCDB searches and builds an index, as well as correlates these data with organ tissue, drug, poison, and disease model research, in order to understand the traceability of the entire process of organs-on-a-chip data from a specific organization to project research to information data in order to achieve comprehensive data. We will also keep working on the synonym system and hope to have it ready for OOCDB as soon as possible. Furthermore, the 3D and mathematical models in OOCDB are a valuable resource for developers and users who develop and use human organ model predictions, effectively promoting the models' safety and accuracy. We will continue to translate the 3D model's content into English. More importantly, dedicated staff will update OOCDB every 3–6 months to include the most recent academic studies and related information. It will also provide the most recent advances in organ-on-a-chip research, analysis of research hotspots, and real-time online statistics in academic-related fields. Therefore, it will provide global on-chip organ researchers and experts in industry and government health medicine with the most comprehensive, real-time, and dynamic latest progress of on-chip organ research. Therefore, our database promotes progress in the field of organs-on-a-chip. Furthermore, due to the serious Corona Virus Disease 2019 (COVID-19) outbreak, we will increase the relevant literature collection on the lung chip and new crown pneumonia epidemic and contribute to the common difficulties.

## Data availability

OOCDB is available at http://www.organchip.cn/.

## Competing interests

The authors have declared no competing interests.

## CRediT authorship contribution statement

**Jian Li:** Conceptualization, Methodology, Software, Validation, Investigation, Resources, Writing – original draft, Writing – review & editing. **Weicheng Liang:** Methodology, Software, Validation, Formal analysis, Investigation, Writing – original draft, Writing – review & editing. **Zaozao Chen:** Methodology, Validation, Formal analysis, Resources, Data curation, Writing – review & editing. **Xingyu Li:** Investigation, Formal analysis. **Pan Gu:** Validation, Writing – review & editing. **Anna Liu:** Validation. **Pin Chen:** Validation. **Qiwei Li:** Resources. **Xueyin Mei:** Investigation. **Jing Yang:** Investigation. **Jun Liu:** Resources. **Lincao Jiang:** Validation, Writing – review & editing. **Zhongze Gu:** Conceptualization, Supervision, Writing – review & editing, Funding acquisition. All authors have read and approved the final manuscript.
